# Effect of Nematogen Doping in Bent-Core Molecular Systems with a Helical Nanofilament and Dark Conglomerate

**DOI:** 10.3390/ma16020548

**Published:** 2023-01-05

**Authors:** Jae-Jin Lee, Suk-Won Choi

**Affiliations:** Department of Advanced Materials Engineering for Information & Electronics, Integrated Education Institute for Frontier Science & Technology (BK21 Four), Kyung Hee University, Yongin 17104, Republic of Korea

**Keywords:** bent-core molecules, helical nanofilaments, dark conglomerates, chirality, nematogen doping

## Abstract

Two types of binary mixtures were prepared. One consisted of a calamitic nematogen and bent-core molecule with a helical nanofilament, whereas the other contained a calamitic nematogen and bent-core molecule with a dark conglomerate. The chiroptical features of these two mixtures were investigated using polarized optical microscopy and circular dichroism. In addition, X-ray diffraction analysis was performed on the two binary mixtures. The chiroptical features of the two mixtures were remarkably different. One mixture showed enhanced chiroptical features, whereas the other did not show chiroptical features. This method may help in distinguishing between helical nanofilaments and dark conglomerates which originate from bent-core molecular systems.

## 1. Introduction

**Figure 1:**
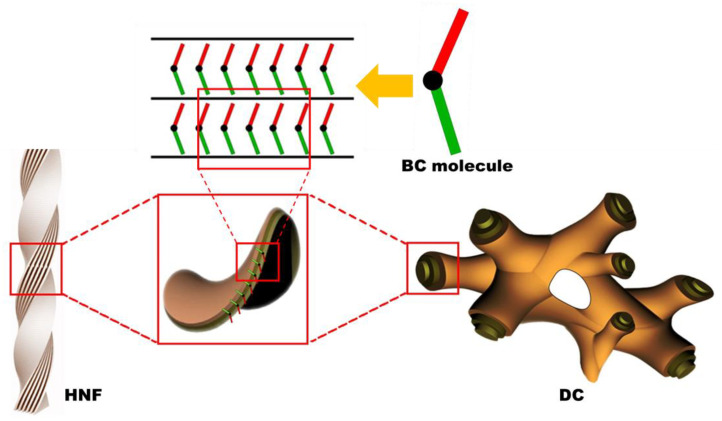
Schematics of the helical nanofilament (HNF) and dark conglomerate (DC) structures. Owing to the intralayer mismatching of the two arms of the molecules and the spontaneous tendency for saddle-splay curvature formation, either HNF or DC structures are assembled [12,16].

Chirality is an interesting topic in the field of liquid crystal science [1,2,3]. Chiral phenomena in achiral bent-core (BC) mesophases have attracted considerable attention since the discovery of spontaneous chiral resolution in achiral BC molecular systems [4,5,6]. The helical nanofilament (HNF) phase is generally observed in the low-temperature range of numerous achiral BC molecular systems. The HNF phase has attracted interest because of its atypical chiral aggregates, in which twisted nanofilaments are formed through the self-assembly of achiral BC molecules [7,8,9], as shown in Figure 1. Although the HNF phase is regarded as a semicrystalline rather than a liquid crystalline (LC) phase, according to recent studies, the spontaneous symmetry-breaking of the HNF phase has been extensively investigated [10,11,12,13,14,15,16].

The dark conglomerate (DC) phase formed by BC molecules has been investigated in recent years [17]. The DC phase exhibits spontaneously chiral-resolved domains with an optical activity similar to that of the HNF phase; however, it appears more isotropic. The morphology of DCs consists of disordered focal conical structures, such as a lyotropic sponge structure, wherein the empty volume is filled with tilted smectic layers (Figure 1). Interestingly, although sponge-like structures exhibit no morphological chirality, the observed macroscopic chirality is attributed to the layered chirality of the tilted smectic layers aggregated by achiral BC molecules [16,17,18,19]. The texture of the DC phase observed via polarized optical microscopy (POM) is smooth and isotropic, whereas the chiral domains of the HNF phase are slightly birefringent. The DC phase often appears in BC molecules with the ferroelectric B2 phase or the B7 phase [20]; however, the factors that cause a particular BC molecule to adopt the HNF or DC phase have still not been thoroughly elucidated [14]. Thus, the DC phase can be erroneously regarded as the HNF phase because the two phases are similar in many cases [16,17,18,19]. Therefore, it would be intriguing to examine the differences in chiroptical features between the HNF and DC phases by disturbing the systems with an external stimulus, such as via mixing with a calamitic nematogen.

When an achiral BC molecule with the HNF phase is blended with a calamitic nematogen, the HNF phase is stabilized. Furthermore, enantiomeric domains become atypically larger (up to a few millimeters in size) than those in the pure HNF phase of the single BC molecule [21]. Subsequent works have elucidated that the phases where enantiomeric domains become atypically larger are the nanoseparated phases between the HNF phase and calamitic nematogen [16,22]. In the nanoseparated phase, where the calamitic nematogen is embedded between the HNF networks, secondary chiral aggregates are generated by the rod-like nematogen affected by the HNF networks. Hence, a significantly high circular dichroism (CD) intensity is observed [21,22]. Binary mixtures consisting of achiral BCs with a DC phase and calamitic nematogens have not been investigated in detail. Therefore, there are few reports on the chiroptical features of binary mixtures.

In this work, we compared the chiroptical features of a binary mixture (M1) composed of a BC molecule with an HNF phase and a calamitic nematogen with that of a mixture (M2) composed of a BC molecule with a DC phase and a calamitic nematogen. Interestingly, the chiroptical feature of M2 was different from that of M1. M2 lost the chiroptical feature because it was mixed with the achiral calamitic nematogen. This was because the addition of the achiral nematogen into the DC phase “diluted” the chirality. Consequently, the CD intensity significantly decreased. This was supported by POM and X-ray diffraction (XRD) analysis.

## 2. Materials

**Figure 2:**
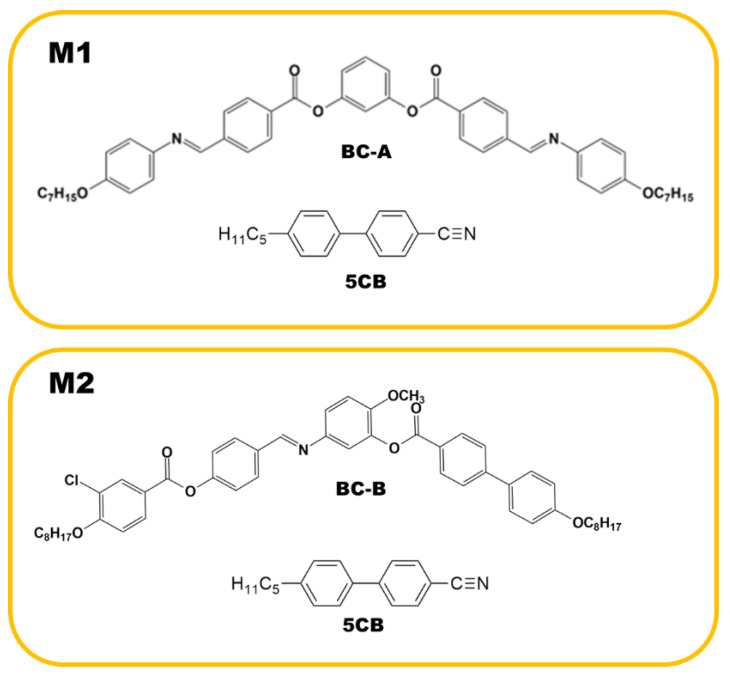
Compositions of M1 and M2 and the chemical structures of BC-A, BC-B, and 5CB.

Two binary mixtures (M1 and M2), consisting of a BC molecule and the rod-like nematogen (5CB), were prepared. One contained BC-A and 50 wt% 5CB, and the other contained BC-B and 50 wt% 5CB. The weighed mixtures were dissolved together in a solvent (chloroform) and mixed using sonication. The mixture was then dried via heating to remove the solvent. The BC molecules were synthesized by our group, and 5CB was purchased from Sigma-Aldrich. The phase sequences of pure BC-A and BC-B (upon cooling) were isotropic (Iso)-172 °C-B2-145 °C-B3-138 °C-HNF and Iso-100 °C-nematic (N)-85 °C-DC, respectively. As shown in the phase sequences, BC-A and BC-B contained the HNF and DC phases in the low-temperature range, respectively. The phase sequence of pure 5CB upon cooling was Iso-34.5 °C-N-22.4 °C-crystal. The compositions of M1 and M2, and the chemical structures of BC-A, BC-B, and 5CB are depicted in Figure 2. 

## 3. Results and Discussion

**Figure 3:**
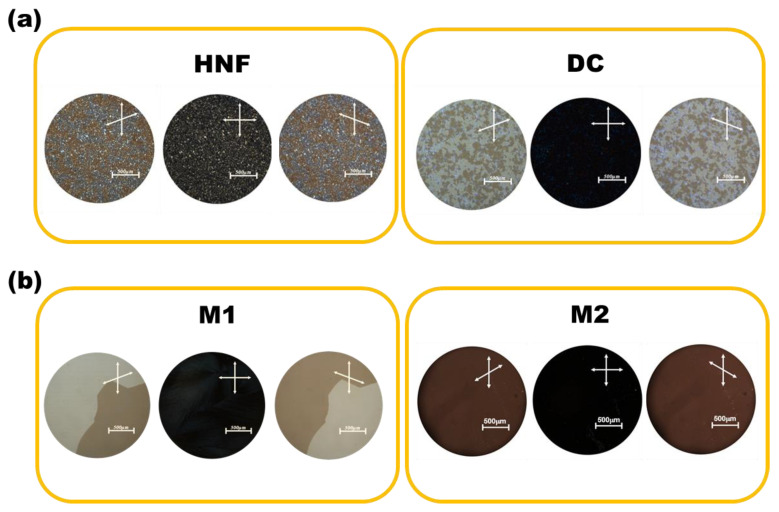
(**a**) Polarized optical microscopy (POM) images of the HNF in pure BC-A and the DC in pure BC-B at 30 °C. (**b**) POM images of M1 and M2 at 30 °C. Scale bar indicates 500 μm.

Figure 3a shows the POM images of the HNF in pure BC-A and the DC in pure BC-B at 30 °C. When one polarizer was slightly rotated counterclockwise, two differently colored domains appeared in the HNF and DC phases. The colors were exchanged when the polarizer was rotated clockwise. This indicated the existence of two enantiomeric domains with almost the same degree of optical rotation but with different signs [16]. In the HNF, the two enantiomeric domains originated from two helical aggregates with opposite helix rotations [16]. The two enantiomeric domains in the DC were due to the layered chirality of the tilted smectic layers within the sponge-like structures aggregated by BC molecules [16,22]. The HNF phase generally showed grainy domains, whereas the DC domains were quite smooth and isotropic. However, it was not easy to distinguish between the HNF and DC through POM observations (Figure 3a). The POM images of M1 and M2 at 30 °C are presented in Figure 3b. The two enantiomeric domains in M1 had grown to a few millimeters in size. This is a well-known feature of mixtures composed of HNFs and rod-like nematogens [22,23,24,25,26,27,28,29]. This state is the nanoseparated phase between the HNF and rod-like nematogen. Only a single domain was observed in M2, and its color did not change with the rotation direction of the upper polarizer. This may indicate that the feature of chirality is lost by mixing the achiral rod-like nematogen with the DC.

**Figure 4:**
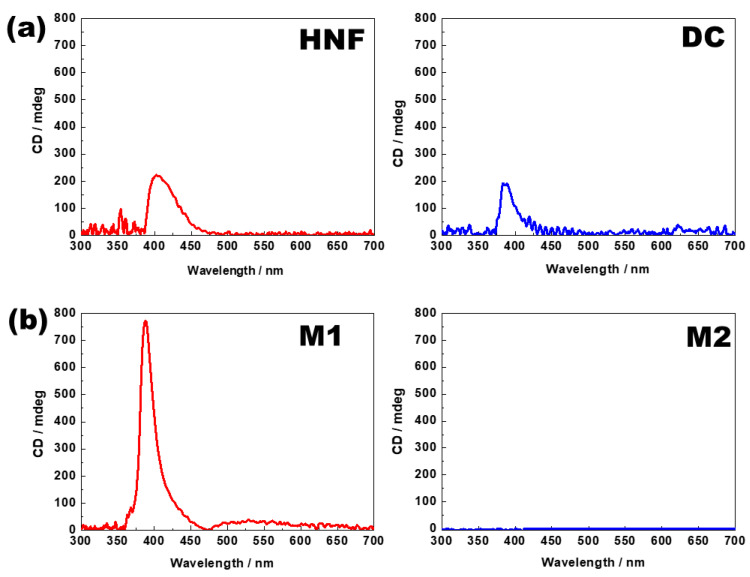
(**a**) Circular dichroism (CD) spectra of HNF in pure BC-A and DC in pure BC-B. (**b**) CD spectra of M1 and M2. All CD spectra were obtained at 30 °C.

A CD spectrometer (J-815, Jasco, Tokyo, Japan) was used to analyze the chiral features of the samples. The samples were injected into a quartz sandwich cell (cell gap ≤ 2 µm), and the CD spectra were investigated in an area with a diameter of 1 mm. Figure 4a shows the typical CD spectra in the HNF of pure BC-A and in the DC phase of pure BC-B. A remarkable CD peak was observed at approximately 400 nm. The typical CD spectra of M1 and M2 at 30 °C are presented in Figure 4b. In M1, the CD intensity increased at approximately 400 nm. This is a typical feature of the nanoseparated phase between the HNF phase and the rod-like nematogen, and it has been reported in numerous binary mixture systems composed of the HNF phase and a rod-like nematogen [21,22,23,24,25,26,27]. In contrast, the CD peak disappeared in M2. This strongly indicated that the chirality was lost in M2. This was because the addition of the rod-like nematogen into the DC phase resulted in a “diluted” chirality.

**Figure 5:**
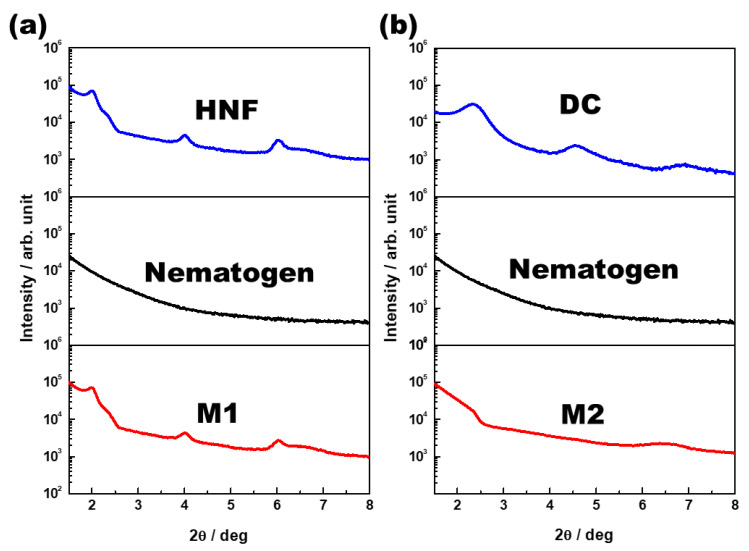
(**a**) X-ray diffraction (XRD) patterns of the pure HNF (BC-A), pure nematogen (5CB), and M1 (at 30 °C). (**b**) XRD patterns of the DC (BC-B), pure nematogen (5CB), and M2 (at 30 °C).

**Figure 6:**
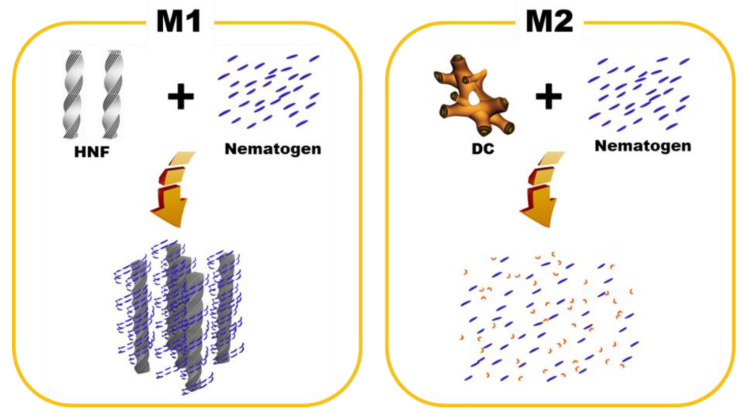
Schematics of the organization principles for M1 and M2.

One-dimensional XRD analysis (Rigaku, Austin, TX, USA) was performed to analyze the organization of M1 and M2. The samples were scanned over a *2*θ range of 1°–8°. The typical XRD patterns of the pure HNF (BC-A), pure nematogen (5CB), and M1 (at 30 °C) are shown in Figure 5a. Multiple peaks for the pure HNF indicated an in-plane crystalline positional order, and the featureless line for the nematogen indicated a liquid-like in-plane order. The XRD pattern for M1 was a simple superposition of those for the pure HNF and the nematogen. As HNF aggregates remained even when the nematogen was mixed with the HNF, M1 was a nanoseparated phase comprising the semicrystalline HNF and LC nematogen. The typical XRD patterns of the DC (BC-B), pure nematogen (5CB), and M2 (at 30 °C) are presented in Figure 5b. The XRD profile of the DC showed broader peaks than that of the typical HNF. Although DCs in numerous BC molecules show featureless XRD patterns, an XRD pattern similar to that of BC-B has been observed for certain BC molecules [23,30]. The XRD pattern of M2 was similar to that of the nematogen, which indicated that the aggregates of the DC were diluted by mixing the nematogen with the DC. Thus, the feature of chirality disappeared. This was because the DC phase was intrinsically close to the LC phase; hence, the DC aggregates were dissolved by the LC nematogen. In contrast, the semicrystalline HNF and LC phases spontaneously underwent nanoseparation. The organizations of M1 and M2, obtained through the XRD analysis, are illustrated in Figure 6.

## 4. Conclusions

We compared two binary mixtures (M1 and M2) consisting of BC molecules and rod-like molecules. M1 contained a nematogen and a BC molecule with an HNF, whereas M2 contained a nematogen and a BC molecule with a DC. Because the HNF and DC phases are similar in many cases, the DC phase can be erroneously regarded as the HNF phase. In this work, we observed that the HNF and DC which originated from BC molecules, which had a low molecular weight, showed different characteristics when mixed with the nematogen. These differences have not been reported to date. The two enantiomeric domains in M1 grew to a few millimeters in size, whereas only a single domain was observed in M2, and its color did not change with the rotation direction of the upper polarizer. In M1, the CD intensity increased at approximately 400 nm, whereas the CD peak disappeared in M2. The XRD pattern for M1 was a simple superposition of those of the pure HNF and nematogen. In contrast, the XRD pattern of M2 was similar to that of the nematogen, which indicated that the aggregates of the DC were diluted by mixing the nematogen with the DC. Thus, the semicrystalline HNF and nematogen spontaneously underwent nanoseparation, whereas the DC was diluted by the nematogen. Hence, nematogen doping may be a powerful method for distinguishing between the DC and HNF phases in BC molecular systems.

## Data Availability

The data presented in this study are available on request from the corresponding author.
